# Comparison of awareness about nephrology and kidney diseases amongst doctors in institutes with and without nephrology departments

**DOI:** 10.12669/pjms.304.4861

**Published:** 2014

**Authors:** Muhammad Anees, Muhammad Ibrahim, Saleem uz Zaman Adhmi, Muhammad Nazir

**Affiliations:** 1 Dr. Muhammad Anees, MBBS, FCPS (Nephrology), Head of Department of Nephrology, Consultant Nephrologist, Shalamar Hospital, Lahore. Pakistan.; 2 Muhammad Ibrahim, Associate Professor of Statistics, Registrar, Govt. M.A.O College, Lahore, Pakistan.; 3 Dr. Saleem Uz Zaman Adhmi, MBBS, FRCP (Medicine), Consultant Physician, Shalamar Hospital, Lahore. Pakistan.; 4 Dr. Muhammad Nazir, Assistant Professor Urology, Department of Urology, Mayo Hospital, KEMU Lahore, Pakistan.

**Keywords:** Awareness, Chronic Kidney Disease, Comparison, Doctors, Nephrology

## Abstract

***Objective:*** To compare the awareness about nephrology and kidney diseases in medical officers and postgraduate trainee doctors working in institutes with and without nephrology departments.

***Methods: ***This cross sectional study was conducted at Nephrology Department, Shalamar Hospital Lahore from 1^st^ Jan to 31^st^ Mar 2013. Doctors working on medical floor with more than one year experience after house job were included in the study. Each doctor was given questionnaire comprising of 28 questions.

***Results:*** Two hundred and eleven doctors fulfilling the criteria were included in the study. Most of the doctors were male 150(71.1%). Knowledge had strong correlation with practice (p= 0.001). Knowledge regarding nephrology and chronic kidney disease (CKD) was found significantly different among doctors of different hospitals [(F=36.09, p=0.000). Practice regarding nephrology and chronic kidney disease (CKD) was found significantly different (F= 7.222, p=0.000)] among doctors of different hospitals of Lahore. Doctors working in the Shaikh Zayed Hospital (SZH), Lahore were having maximum score in the components of the knowledge and practice than other hospitals.

***Conclusion: ***Awareness of the Medical Officers and postgraduate doctors is poor regarding nephrology and kidney diseases. Doctors working in institutes with established nephrology services have better knowledge than other units. Working experience of doctors improve the practice significantly.

## INTRODUCTION

Nephrology is a specialty which deals with medical diseases of kidneys. In Pakistan Professor SA Jaffar Naqvi was 1^st^ Nephrologist who joined in Jinnah Postgraduate Medical Center Karachi in 1970.^1^ After that in 1980’s postgraduate training in nephrology was started by College of Physician and Surgeon of Pakistan in Lahore, Karachi & Peshawar and by the end of 2000 there were 12 nephrology centers for postgraduate academic affiliation. But in the last couple of years there is tremendous improvement in accreditation of institutes for training in nephrology and now there are about 28 centers recognized for training in nephrology.^2^ At present, there are only about 80 formally trained nephrologists in Pakistan for a population of about 160 million as compared to the United States which has more than 5000 nephrologists for a population of about 300 million.^3^ The underdevelopment of nephrology as specialty leads to poor training and teaching of doctors. This leads to lack of awareness for the early detection and management of kidney diseases amongst doctors.^4^ Lack of knowledge about clinical practice guidelines and the referral of the patients with chronic kidney disease (CKD) to nephrologist is at very late stage which increases morbidity and mortality of dialysis patients.^5^^,^^6^ Even the training of physicians in developed countries is not up to the mark.^7^^,^^8^

There is very limited data in Pakistan about the knowledge and practice of kidney diseases amongst doctors and family physicians. But up till now no study has been conducted to compare the level of education between doctors working in hospitals with without nephrology departments. So this study was conducted to determine the difference in knowledge and practice of doctors between the institute with and without the established nephrology departments.

## METHODS

This cross sectional study was conducted in different tertiary care teaching hospitals of Lahore [(Mayo Hospital (MH), Sir Ganga Ram Hospital (SGRH), Services Institute of Medical Sciences (SIMS), Fatima Memorial Hospital (FMH), Lahore General Hospital (LGH), Shalamar Hospital (SH), Jinnah Hospital (JH), Shaikh Zayed Hospital (SZH)] in institutes with and without nephrology departments. Doctors working on medical floor with more than one year experience after house job were included in the study. Doctors who were medical officers and postgraduate trainees in medicine were included for assessment. Doctors working in others specialties and nephrology were excluded. Each doctor was given questionnaire comprising of 28 questions. This questionnaire was having three components covering Knowledge, Attitude and Practice regarding nephrology and kidney diseases. The content of questionnaires was covering the basic concepts about the kidneys diseases. Each participant was given 10 to 15 minutes for completing the question at the spot and identification of the doctors was kept secret.

***Statistical*** ***analyses: ***The data was entered and analyzed by using standard SPSS software version-17 (SPSS Inc, Chicago) for statistical analysis. Continuous variables were expressed in the form of Mean ± SD. To signify the gap among three variables like knowledge, attitude and practice, One way ANOVA was used. *P *value of <0.05 was considered significant. Crude Knowledge score (0-9), Attitude (0-5) and Practice (0-14) was standardized in terms of 100. Correlation was used to determine the relation between different variables.

## RESULTS

**Fig.1:**
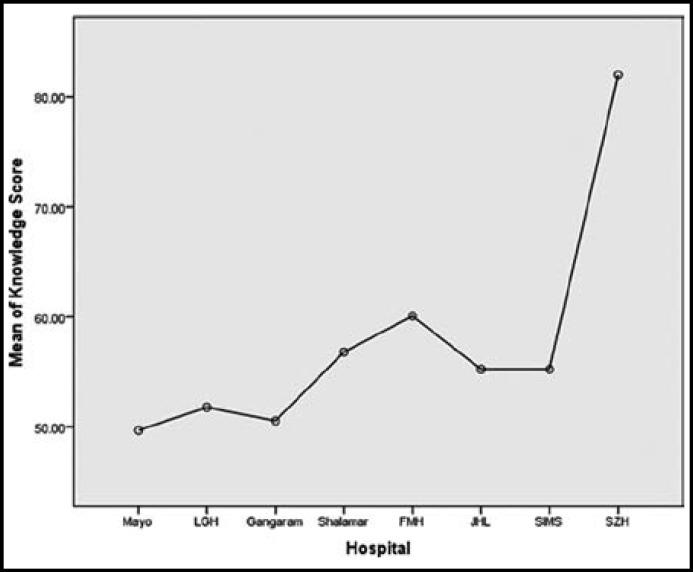
Graph showing mean knowledge score of doctors working at different hospitals of Lahore

**Fig.2:**
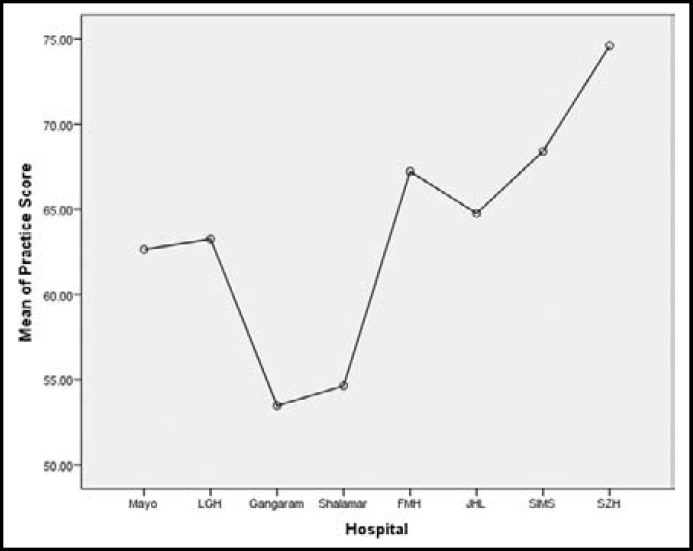
Graph showing mean practice score of doctors working at different hospitals of Lahore

**Fig.3:**
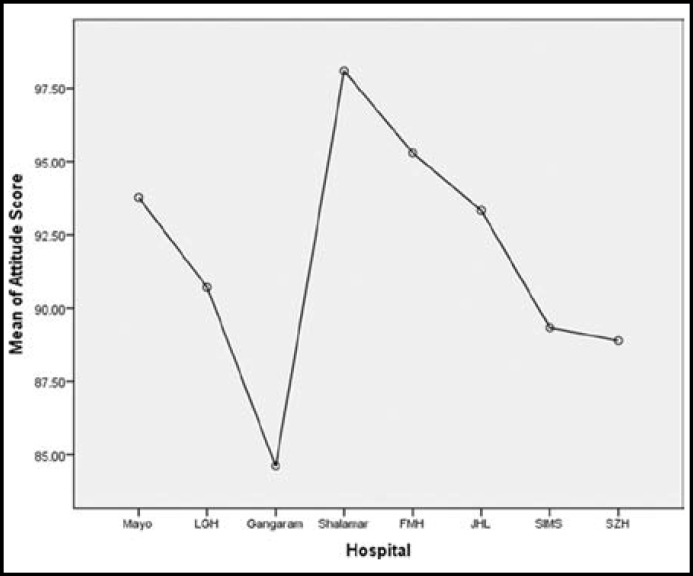
Graph showing mean attitude score of doctors working at different hospitals of Lahore

Two hundred and eleven doctors fulfilling the criteria were included in the study. Numbers of the doctors of the different hospitals is shown in the Table-I. Maximum number of the doctors were from MH who participated in the study. Most of the doctors were males 150(71.1%) and rest were females 61(28.9%). Mean working experience of the doctors after graduation was 29 weeks with range of one to one twenty weeks. Knowledge was having strong correlation with practice (p= 0.001) while there was no correlation between knowledge & attitude and practice & attitude (p=0.774, p=0.443). Knowledge and practice regarding nephrology and CKD was found significantly different among doctors of different hospitals [(F=36.09, p=0.000), (F= 7.222, p=0.000)] of Lahore respectively. Doctors working in the SZH were having maximum score in the components of the knowledge and practice than doctors of other hospitals as shown in Fig. 1 & 2. Component of the attitude was maximum in SH than all the hospitals of Lahore as shown in Fig.3. Working experience and practice score regarding nephrology and CKD was having significant correlation (p=.005) but knowledge and attitude did not.

## DISCUSSION

Prevalence of CKD is about 150 patient/million population/year.^9^ According to a local study by Jafar TH, about 15-20 percent of persons, 40 years of age or older have a reduced kidney function.^10^ According to 1990-1994 National Health Survey one third of Pakistani at the age of 45 years or above have hypertension.^11^ In Pakistan health system is very week and only 4% of GDP is spent on health.^12^ Being under developed country more stress of the health system is on infectious and communicable diseases like Malaria, Typhoid, Cholera, Dengue, Polio etc. Diseases which are increasing with urbanization are not being focused especially CKD which is exploding day by day. Nephrology services are in early phase of establishment in teaching institutes with the result that there is drastic increase in such diseases. Due to non availability of nephrologists and nephrology services even at DHQ hospitals, patients have to travel a lot seeking consultation which further makes the things worse.

**Table-I:** Number of the doctors from different hospitals

***S. No***	***Name of Institute***	***No (%)***
1	Mayo Hospital (MH)	42(20)
2	Jinnah Hospital (JH)	31(14)
3	Services institute of Medical Sciences (SIMS)	27(13)
4	Shaikh Zayed Hospital (SZH)	25(12)
5	Sur Ganaga Ram Hospital (SGRH)	25(12)
6	Lahore General Hospital (LGH)	25(12)
7	Shalamar Hospital (SH)	19(9)
8	Fatima Memorial Hospital (FMH)	17(8)

In this study doctors working at SZH were having maximum knowledge and practice score than all the doctors working in the teaching tertiary care hospital of Lahore. In 1980’s SZH Lahore and Pakistan Institute of Medical Sciences Islamabad, were only two center which were affiliated for post graduate training in nephrology. Nephrology department Shaikh Zayed Hospital was started in 1986 by Prof. Tahir Shaffi. This was only institute which played a pivotal role in training doctors in nephrology in lower and middle Punjab. This institute not only produced nephrologist but played a commendable role in creating awareness about kidney diseases in the general population. The better knowledge and practice score of the KAP survey shows the advantages of established nephrology department as compared to all other teaching institute in the Lahore where nephrology departments are in infancy stage. In Lahore five departments have been accredited for post graduation in nephrology in the last 3-4 years. But up till now there is no advantages of these department as these are in different phases of development. These departments are having shortage of faculty and facilities as well. There is need to promote these department by government to allocate more and more funds exclusively for them. Without promoting these departments and producing more nephrologists explosion of CKD cannot be stopped. These department will train more general physicians and general practitioner (GP’s) for creating awareness for early detection and prevention of kidney disease. 

In this study knowledge and practice had strong correlation. It means doctors who were having knowledge they were using it practically as well, because in this study the response of doctors to knowledge related questions was counter checked by questions in the practice segment of KAP survey. The correlation is also provided by other studies.^13^ There is no correlation of knowledge and practice with attitude. The doctors of all the institutes got maximum score in attitude segment of KAP survey except SGRH. The doctors working on medical floor of all the hospital were having positive attitude toward learning, creating awareness and establishing separate nephrology department in their institute. Majority of the doctors feel that nephrology should be taught during graduation and house job. Due to the shortage of nephrologists in the country most of the patients present to doctors working in medicine and other specialties. So by teaching physicians and creating awareness for early detection, management of kidney diseases will compensate the lack of nephrologists. By teaching and guiding the doctors about early referral to nephrologists will reduce the morbidity and mortality of CKD patients.^14^^,^^15^

Working experience of the doctors in this study was having significant affect on the daily practice about nephrology and CKD. As doctors become senior and gain more experience their practice improves regarding the detection and management of CKD. But working experience does not affect the knowledge and attitude significantly as was shown in this study.

## CONCLUSION

Knowledge of the Medial Officers and postgraduate doctors is poor regarding kidney diseases. Doctors working in institutes with nephrology services have better knowledge than other units. Knowledge and practice has strong correlation. Working experience of the doctors improves the practice. There is need to establish full fledge nephrology departments at teaching hospitals with immediate effect and make essential arrangement for three months rotation in nephrology in fellowship program of medicine. Renal medicine must be taught during graduation as separate subject. There should be continuing medical education (CME) programs for GP’s on regular basis.

## References

[1] Naqvi SAJ (2000). Dialysis and Transplantation News Nephrology services in Pakistan. Nephrol Dial Transplant.

[2] http://www.cpsp.edu.pk/index.php?code=YWNjcmVkaXRlZHxhY2NyZWRpdGF0aW9uLnBocHwwfEZDUFN8MA.

[3] Jafar TH (2006). The growing burden of CKD in Pakistan. NEJM.

[4] Stack AG (2003). Impact of timing of nephrology referral and pre-ESRD care on mortality risk among new ESRD patients in the United States. Am J Kidney Dis.

[5] Anees M, Mumtaz A, Ibrahim M, Shaheen SM, Asghar A (2010). Effect of Anemia and Hyperhomocysteinemia on mortality of patients on Hemodialysis. Iranian JKD.

[6] Anees M, Mumtaz A, Nazeer M, Ibrahim M, Rizwan SM, Kausar T (2007). Referral pattern of hemodialysis patients to nephrologists. J Coll Physicians Surg Pak.

[7] Campbell JD EB, Hosokawa M, Van Stone JC (1989). The timing of referral of patients with end-stage renal disease. Nephrol Dial Transplant.

[8] Fox CH, Brooks A, Zayas LE, McClellan W, Murray B (2006). Primary care physicians’ knowledge and practice patterns in the treatment of chronic kidney disease: An Upstate New York Practice-based Research Network (UNYNET) study. J Am Board Fam Med.

[9] Naqvi SAJ (2009). Renal diseases in Pakistan. “Time to Act” JNRT.

[10] Jafar TH, Hatcher J, Chaturvedi N, Levey AS (2005). Prevalence of reduced estimated GFR (eGFR) in Indo Asian population. J Am Soc Nephrol.

[11] https://www.google.com.pk/?gws_rd=cr&amp;ei=jVjJUpqAEMqPtAbPqoCQDQ#q=national+health+survey+of+pakistan+1990+94.

[12] https://www.google.com.pk/search?q=Highlights+of+the+Pakistan+Economic+Survey+of+Pakistan+2012-2013.&amp;oq=Highlights+of+the+Pakistan+Economic+Survey+of+Pakistan+2012-2013.&amp;aqs=chrome..69i57.7095j0j9&amp;sourceid=chrome&amp;espv=210&amp;es_sm=93&amp;ie=UTF-8.

[13] Kanwal A, Asim M, M Ibrahim, Toqeer A, Sajid AQ (2012). Knowledge, Attitude and Practice of Quality Assurance Among Medical Laboratory Technologists Working in Laboratories of Lahore. Physicians Academy.

[14] Farooq Z, Mehmood A, Saeed S, Raja KM (2010). Early versus late arterio venous fistulae: impact on failure rate. J Ayub Med Coll Abbot.

[15] Kim do H, Kim M, Kim H, Kim YL, Kang SW, Yang CW Early Referral to Nephrologist Improved Patients Survival: Prospective Cohort Study for End-Stage Renal Disease in Korea.

